# Potential Use of Colored LED Lights to Increase the Production of Bioactive Metabolites *Hedyotis corymbosa* (L.) Lam

**DOI:** 10.3390/plants11020225

**Published:** 2022-01-15

**Authors:** Anh Tuan Le, Ju-Kyung Yu, Gyung-Deok Han, Thuong Kiet Do, Yong-Suk Chung

**Affiliations:** 1 Department of Plant Resources and Environment, Jeju National University, Jeju 63243, Korea; anhtuan.sinhhoc@gmail.com (A.T.L.); hangds@jejunu.ac.kr (G.-D.H.); 2 Department of Plant Physiology, Faculty of Biology—Biotechnology, University of Sciences, VNU-HCM, Ho Chi Minh 70000, Vietnam; 3 Seeds Research, Syngenta Crop Protection LLC, Research Triangle Park, 9 Davis Dr, Durham, NC 27703, USA; ju-kyung.yu@syngenta.com

**Keywords:** *Hedyotis corymbosa* (L.), light-emitting diode (LED), plant growth, photosynthesis, metabolites production

## Abstract

*Hedyotis corymbosa* (L.) Lam is a wild herb that is used in traditional Indian, Chinese, and African medicine. Light-emitting diode (LED) technology is paving the way to enhance crop production and inducing targeted photomorphogenic, biochemical, or physiological responses in plants. This study examines the efficiency of *H. corymbosa* (L.) Lam production under blue 450 nm and red 660 nm LED lights for overall plant growth, photosynthetic characteristics, and the contents of metabolite compounds. Our research showed that blue LED lights provided a positive effect on enhancing plant growth and overall biomass. In addition, blue LED lights are more effective in controlling the production of sucrose, starch, total phenolic compounds, and total flavonoid compared to red LED lights. However, blue and red LED lights played essential but different roles in photosynthetic characteristics. Our results showed the potential of colored LED light applications in improving farming methods and increasing metabolite production in herbs.

## 1. Introduction

*Hedyotis corymbosa* (L.) Lam, a synonym of *Oldenlandia corymbosa* (L.) Lam, is a wild herb in the Rubiaceae family and is also an annual plant that can be erect or prostrate and is sparsely branched. It is widely distributed throughout the tropical regions of America, Africa, Asia, and the islands of the western Pacific [1]. It is a popular homeopathic medicine in India, China, and Africa and is harvested from the wild for local use as food, medicine, and a natural dye. The whole plant extract contains high levels of antioxidant activity due to secondary compounds such as phenolics and flavonoids [2,3], which are known for their anti-inflammatory, antibacterial, antioxidant, and anti-cancer properties [4,5,6,7].

The sustainable, high-yielding number of secondary compounds is the utmost goal in the herb industry. Various technologies are applied to pursue it, such as tissue and cell culture, precursor feeding, and elicitation treatments [8]. The treatments, such as using methyl jasmonate (elicitors), sugar (induction of osmosis), polyethylene glycol (PEG) (drought stress) on cell suspension, and shoot clusters in vitro, were applied to increase the contents of secondary compounds in *H. corymbosa* (L.) Lam [9,10,11]. Recently, genome editing technology using CRISPR/Cas9 edits is being introduced to boost the production of secondary metabolites [12]. However, there are a few challenges with these technologies, such as difficulties in large-scale implementation, the limitation of technology accessibility, and poor the social acceptance of gene-edited plants [13,14]. Herb producers are continuing to explore more reliable and sustainable technologies to increase the yield of secondary metabolites without genetic manipulation and chemical intervention.

Light is essential for the phytochemical synthesis and the accumulation of secondary metabolite compounds in plants [15,16,17,18]. LED lights have narrow bandwidths, produce less heat, and are a convenient integration into electronic systems. LED lights are applicable for plant light experimentations to study the influence of light in photosynthetic performance, plant growth, and physiological reactions [19]. In the visible light spectra, blue and red light energy was demonstrated as having a more significant effect than other photosynthetically active radiation (PAR) wavelengths [20,21]. Changes in LED wavelengths and light intensity can shift the primary and secondary metabolism in plants, alter the plant’s metabolism, and effect the accumulation of the plant’s functional products [16]. Several studies have shown that applying LED lights in the right spectrum in plant cultivation can increase the total amount of phenolic compounds and flavonoids in plants [17,18].

The goal of this study was to investigate the efficiency of *H. corymbosa* (L.) Lam production under blue 450 nm and red 660 nm LED lights for overall plant growth, photosynthetic characteristics, and the contents of some key secondary metabolites.

## 2. Results

The experiments of *H. corymbosa* (L.) Lam were conducted under three different light conditions: monochromatic red LED 660 nm (R), blue LED 450 nm (B), and a fluorescent lamp (FL). Table 1 shows that the blue LED light caused the largest reduction in the total leaf area, followed by the red LED light and FL. The blue LED light had the highest effect on stomatal aperture, Hill reaction activity, and respiratory rate comparatively, but no different effects were detected between the red LED light and FL (Table 1). In general, various studies demonstrated that both blue and red LED lights contributed to enhanced plant growth [22,23,24], but our study showed the blue LED light was more effective than the red LED light for *H. corymbosa* (L.) Lam.

The values of F_v_/F_m_, qP, and ETR of the *H. corymbosa* (L.) Lam under the blue LED light were significantly higher (*p* ≤ 0.05) than those under the red LED light, while the value of qN under the red LED light was higher (Table 2). Notably, the value of ETR under the blue LED light was almost threefold higher than that under the red LED light. This result aligned with the research by Miao et al. (2016), which proved that the blue LED light is more effective in ETR compared to the red LED light [25]. The contents of three types of pigments (chlorophyll a, chlorophyll b, and carotenoid) were measured to investigate how each pigment responds to different light sources (Table 3). There was no significant difference found in the content of chlorophyll a under three different light sources. However, the blue LED light decreased the content of chlorophyll b, while the red LED light decreased the content of carotenoid. Consequently, the a/b ratio and the (a + b)/c ratio were different depending on the light sources, which suggests that optimal LED light sources could be selected for plant cultivation.

Biomass was also measured in different light sources (Table 4). Compared to the control, the blue LED light increased the fresh weight of *H. corymbosa* (L.) Lam, while the red LED light reduced the fresh weight of *H. corymbosa* (L.) Lam. However, only the blue LED light increased the dry weight, and there was no dry weight difference measured between the red LED light and FL. In conclusion, the blue LED light was only effective in increasing both fresh and dry weight. Most studies demonstrated both blue and red LED lights contributing to enhanced overall plant biomass [26,27,28]; however, it was not applicable for *H. corymbosa* (L.) Lam.

Table 5 demonstrated the difference in metabolite production under different light sources. Four compounds were measured: sucrose, starch, total phenolic compounds, and total flavonoid. In both blue and red LED lights, sucrose contents decreased while starch content was increased under the blue LED light. In total phenolic compounds, both blue and red LED lights were effective in production, and the red LED light doubled the amount of the total phenolic compounds compared to the control. The blue LED light was effective in total flavonoid production, but there was no difference measured between the red LED light and control. Therefore, the blue LED light was more effective in the production of sucrose, starch, total phenolic compounds, and total flavonoid (Table 5).

## 3. Discussion

Sucrose, as the product of photosynthesis through the Calvin cycle, is the main organic source [29]. It could be stored as starch, transported to be used for metabolism, or be used for respiration to generate the energy source ATP. The stored starch is responsible for biomass, although starch could be converted to sucrose [30,31]. Respiration produces ATP as an energy source to synthesize phenolic and flavonoid compounds [29,32]. Therefore, increasing plant biomass and the total amount of metabolite compounds is important for plant cultivation, especially for *H. corymbosa* (L.) Lam, which is used as a health supplement in various applications. This study demonstrated that the most effective light source to make total phenolic and flavonoid compounds is the blue LED light, although the conversion ratio of sucrose/starch and metabolic compounds in *H. corymbosa* (L.) Lam from different light sources is not known. Further study to explain the detailed mechanism of conversion from a photosynthesis product to phenolics and flavonoids is needed.

The decrease of sucrose in LED light treatment was correlated with an increase of total phenolic content in leaves of *H. corymbosa* (L.) Lam ex vitro plants. According to Darko et al. [16], light not only changes the quality of photosynthesis products but also affects secondary metabolism. The same trend was recorded in *Brassica napus L*., *Lactuca sativa*, *Ocimum basilicum*, and *Rhodiola imbricata* when the plants were exposed under red or blue LED with light intensity from 50 to 200 μmol·m^−2^·s^−1^ [15,33,34,35]. Previous studies have shown that total phenolic compound was increased with additional monochromatic LED light, even though the blue or red LED has been applied only as supplemental light or combine with another light source [17,18]. Several studies have reported the relationship between red or blue LED and phenylalanine ammonia-lyase (PAL), which is the key enzyme in the phenolic metabolic pathway [36,37,38].

## 4. Materials and Methods

### 4.1. Plant Material and Growth Conditions

For the in vitro experiment, 10-day-old *H. corymbosa* (L.) Lam plants grown from seeds on MS medium with 30 g/L of sugar were used. The pH of the medium was adjusted to 5.8 before gelling the medium with 6 g/L of agar. The culture conditions were controlled at 27 ± 2 °C with a relative humidity of 65 ± 5%, 50 μmol·m^−2^·s^−1^ using a fluorescent lamp with a 12 hours of light and 12 hours of dark (12/12) photoperiod. For ex vitro treatments, plants with two pairs of true leaves were grown from a seed on clean soil and cow manure (ratio 3:1) in a greenhouse under 450 ± 100 μmol·m^−2^·s^−1^ sunlight at 32 ± 2 °C with a relative humidity of 70 ± 5%.

### 4.2. Light Treatment

**Figure 1:**
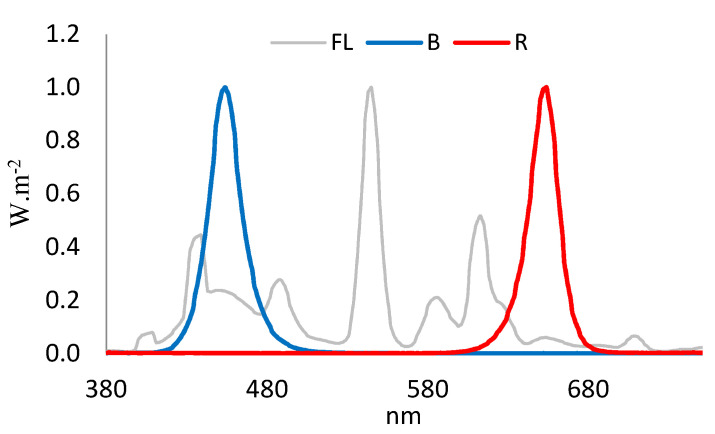
Spectral composition in different light treatments: blue (B), red (R) light-emitting diodes (LEDs), and fluorescent light (Fl). Peak wavelength numbers are shown for each broadband.

LED light tubes were provided by the LED Agri-Bio Fusion Technology Research Center (LAFTRC) at Chonbuk National University in the Republic of Korea. The experiments were conducted in three treatments: monochromatic red LED 660 nm (R), blue LED 450 nm (B), and a fluorescent lamp (FL) (Philips, The Netherlands) as a control. The emission spectra from light sources were measured with an MK-350S (UPRtek, Taipei, Taiwan) (Figure 1).

The in vitro and ex vitro plant materials were incubated for four weeks under light treatment with a 12/12 photoperiod, and a light intensity was controlled at 100 μmol·m^−2^·s^−1^ and measured by a LI-250A with a LI-190R Quantum Sensor (LI-COR Inc., Lincoln, NE, USA) above the plant canopy.

### 4.3. Measurements

Measurements of the leaf area: Leaves were separated from plants, and the leaf area was determined by imaging (Canon IXUS 220HS, Monterey, CA, USA) and analysis using LIA for Win32 software. The area of a single leaf was calculated as the ratio of total leaf area to leaf number.

Measurements of stomatal aperture: The fifth leaves (from the top of the plants) were swept onto the underside with a cyanoacrylate glue (mixed in toluene and ethyl acetate solvents) and fixed on the slide. The surface of the leaf with the stomata was printed on the cyanoacrylate film [39]. The cyanoacrylate film was photographed by a CKX41 inverted microscope (Olympus, Tokyo, Japan) with a DFC450 camera (Leica, Wetzlar, Germany), and the stomatal aperture was measured by the ImageJ program (Wayne Rasband, imagej.net).

Isolation of chloroplasts and determination of the Hill reaction activity: The Hill reaction activity was assessed and described by Henselová et al. (2003), with slight modifications [40]. To isolate chloroplast, 0.5 g of mature leaves were ground in a mixture of 9 mL NaCl 0.35 M and 1 mL Tris 50 mM with a pH of 8. The extract mixture was centrifuged at 500 rpm (five minutes), and the supernatant was collected. The supernatant was further centrifuged for a second time at 2000 rpm (five minutes), and the residue collected contained isolated chloroplasts. The manipulations were performed at 3−5 °C in the dark. The chloroplast density was determined using a Neurban erythrocyte counting chamber. Half a milliliter of chloroplast suspension that was isolated from the leaves determined the Hill reaction activity through the color loss of 2,6-dichlorophenol indophenol 0.25 × 10^−4^ M (DCIP) by optical density at 600 nm (GENESYS™ 30, Thermo Fisher Scientific Inc, Waltham, MA, USA) in the phosphate buffer, which consisted of 0.15 M Na_2_HPO_4_·12 H_2_O and 0.15 M KH_2_PO_4_ (8:2) (pH 6.5) after 10 min of being exposed to growth light.

Measurements of net respiratory rate: The pair of the fifth leaves were isolated and measured using the gas exchange rate by an oxygen electrode with the LD2 electrode chamber of the Leaf Lab 2 system (Hansatech Instruments, Hansatech, United Kingdom). The leaf chamber temperature was controlled at 27 ± 1 °C. The net respiratory rate of the sample, in dark conditions, was calculated on the basis of the amount of oxygen absorbed in the leaf chamber (μmol O_2_·m^−2^·s^−1^).

Measurements of chlorophyll fluorescence parameters: A PAM-2500 portable chlorophyll fluorometer (Heinz Walz GmbH, Effeltrich, Germany) was used for chlorophyll fluorescence parameter measurements of the *H. corymbosa* (L.) Lam leaves. Briefly, the fifth leaves were dark-adapted 15 min before the measurement was taken by using dark leaf clips DLC-8. The leaf sample recorded the minimum fluorescence (F_o_) under modulated light (0.1 μmol·m^−2^·s^−1^), and, subsequently, maximum fluorescence (F_m_) was determined by using a saturation pulse of red light (5700 μmol·m^−2^·s^−1^, 0.8 s duration). The leaf was exposed to the treatment light for 10 min, and then the maximum fluorescence value (F_m_′) after a saturation pulse of red light (5700 μmol.m^−2^·s^−1^, 0.8 s duration) and the minimum fluorescence value (F_o_’) in 5 s in far-red light were recorded. The parameters of chlorophyll fluorescence included maximal quantum yield of photosystem II (F_v_/F_m_, from 0 to 1), photochemical fluorescence quenching coefficient (qP), non-photochemical fluorescence quenching coefficient (qN), and relative electron transfer rate (ETR), which were measured and calculated automatically as follows [41].

The maximal quantum yield of photosystem II:

$$Y\left(\mathrm{II}\right)=\mathrm{Fv}/\mathrm{Fm}=\frac{\mathrm{Fm}-\mathrm{Fo}}{\mathrm{Fm}}$$

The photochemical fluorescence quenching coefficient:

$$\mathrm{qP}=\frac{{\mathrm{Fm}}^{\prime }-F}{{\mathrm{Fm}}^{\prime }-{\mathrm{Fo}}^{\prime }}$$

The non-photochemical fluorescence quenching coefficient:

$$\mathrm{qN}=1-\frac{{\mathrm{Fm}}^{\prime }-{\mathrm{Fo}}^{\prime }}{\mathrm{Fm}-{\mathrm{Fo}}^{\prime }}$$

The electron transfer rate:

ETR=PAR×ETRFactor×PPS2PPPS×Y(II)

PAR was light intensity measured from treatment light. The ETR_Factor_ value was 0.84, and the P_PS2_/P_PPS_ value was 0.5.

Determination of fresh and dry weight: All leaves were separated from the plant, and the fresh weight was determined by HR-202i balance (A&D Company, Limited, Japan). For dry weight determination, the leaves were dried in an electric drying oven (UNB 500, Memmert, Germany) at 60 °C for three days until a constant mass was achieved.

### 4.4. Compound Extraction

Extract and determine total sugar and starch content: The fresh leaf samples were ground in absolute ethanol, centrifuged, and the supernatant (supernatant 1) was collected. The residue was hydrolyzed by perchloric acid, centrifuged, and the supernatant (supernatant 2) was also collected. The optical density of the mixture of the two supernatants (separately) with phenol and sulfuric acid was measured. The extraction and determination of total sugar and starch content were based on the description of Coombs et al. [42].

Extract and estimate total phenolic and flavonoid: Total phenolic and flavonoid extraction was carried out using the method of Victório et al. (2009) [43]. Briefly, a sample of 1 g of dry leaves was extracted with 20 mL of ethanol 70%, and the microwave method was used (Panasonics, auto sensor diet, full power) at 60 °C and filtered through 0.45 μm filter paper. The total phenolic in the leaves was estimated by the Folin–Ciocalteu method according to the principle of the reduction of the Folin–Ciocalteu reagent by the phenol compound in an alkaline medium and the resulting color product. The phenolic content was calculated as gallic acid equivalents (GAE) per gram of fresh weight leaves by optical density at 765 nm and the gallic acid calibration curve. The total flavonoid content was determined by the aluminum chloride colorimetric method. The total flavonoid content was calculated as rutin equivalents (RE) per gram by optical density at 510 nm and the rutin calibration curve [44].

### 4.5. Statistical Analysis

All experiments have six replications per treatment. The data recorded from the experiments were statistically processed using SPSS 11.5 for Windows. Statistical significance was estimated at *p* < 0.05 according to *t*-test and Duncan’s multiple range test with one-way ANOVA. All data were given as mean ± SE.

## 5. Conclusions

Plants can make a lot of valuable chemical compounds from various metabolic processes. Those compounds were collected by growing plants and harvesting the target organs that contain them. With advent of new technology, scientists use genetic approaches to increase the target product by manipulating genes. However, these methods can be controversial. However, in the current study, the different photosynthesis parameters of *H. corymbosa* (L.) Lam under monochromatic blue or red LED light was investigated. A total of 450 nm blue LED promoted photosynthesis in leaves through an increased stomatal aperture; the mechanisms to protect the photosynthetic apparatus were maintaining the carotenoid content and increasing the appearance of starch in leaves. Along with this, blue LED light increased the respiratory rate, thus leading to the greater biomass of plants. In contrast, the plants that were exposed long term under red LED light (660 nm) treatment showed the “red light syndrome” specifically symptom with the lower biomass of plants. Both monochromatic LED lights play an important role in controlling the distribution of photosynthetic products in secondary compound metabolism such as total phenolic and total flavonoids in leaves of *H. corymbosa* (L.) Lam. Understanding how plants respond to different light sources makes it possible to control the growth and accumulation of desired bioactive compounds in plants.

## Figures and Tables

**Table 1:** The leaf area, stomatal aperture, Hill reaction activity, and respiratory rate of *H. corymbosa* (L.) Lam leaves after 4 weeks of being grown under different light sources at 100 μmol·m^−2^·s^−1^ light intensity.

Light Source	Leaf Area (cm^2^)	Stomatal Aperture (µm)	Hill Reaction Activity (nmol DCIP.Million of Chloroplast^−1^·min^−1^)	Respiratory Rate (µmol O_2_·cm^−2^·min^−1^)
Fluorescent lamp	1.64 ± 0.08 ^a^	3.30 ± 0.08 ^b^	0.087 ± 0.001 ^b^	0.145 ± 0.02 ^b^
Blue LED	1.13 ± 0.05 ^c^	4.22 ± 0.17 ^a^	0.108 ± 0.002 ^a^	0.219 ± 0.02 ^a^
Red LED	1.39 ± 0.05 ^b^	3.30 ± 0.06 ^b^	0.083 ± 0.001 ^b^	0.166 ± 0.01 ^b^

Means of ± standard errors followed by different letters within columns are significantly different by Duncan’s multiple range test with one-way ANOVA; *n* = 6.

**Table 2:** The maximal quantum yield of PSII (F_v_/F_m_), photochemical fluorescence quenching coefficient, non-photochemical fluorescence quenching coefficient, and electron transfer rate of *H. corymbosa* (L.) Lam leaves after 4 weeks of growth under different light sources at 100 µmol·m^−2^·s^−1^ light intensity.

Light Source	F_v_/F_m_	qP	qN	ETR (µmol Electron·m^−2^·s^−1^)
Blue LED	0.70 ± 0.01	0.87 ± 0.02	0.20 ± 0.02	24.48 ± 0.53
Red LED	0.58 ± 0.02 *	0.45 ± 0.04 *	0.38 ± 0.02 *	9.15 ± 0.43 *

* Significant at *p* ≤ 0.05; *n* = 6.

**Table 3:** The pigments content in leaves of *H. corymbosa* (L.) Lam after 4 weeks of growth under different light sources at 100 µmol·m^−2^·s^−1^ light intensity.

Light Source	Pigment Content (mg/g _FW_)			a/b Ratio	(a+b)/c Ratio
	Chlorophyll a	Chlorophyll b	Carotenoid		
Fluorescent lamp	2.05 ± 0.09 ^a^	0.54 ± 0.03 ^a^	0.74 ± 0.03 ^a^	3.77 ± 0.03 ^b^	3.56 ± 0.03 ^b^
Blue LED	2.04 ± 0.02 ^a^	0.48 ± 0.01 ^b^	0.74 ± 0.02 ^a^	4.25 ± 0.02 ^a^	3.47 ± 0.08 ^b^
Red LED	2.09 ± 0.05 ^a^	0.57 ± 0.02 ^a^	0.68 ± 0.01 ^b^	3.70 ± 0.03 ^b^	3.93 ± 0.11 ^a^

Means of ± standard errors followed by different letters within columns were significantly different according to Duncan’s multiple range test with one-way ANOVA; *n* = 6.

**Table 4:** Fresh and dry weight of *H. corymbosa* (L.) Lam plants after 4 weeks of being grown under different light sources at the same light intensity at 100 µmol·m^−2^·s^−1^.

Light Source	Fresh Weight (g)	Dry Weight (g)
Fluorescent lamp	21.13 ± 0.33 ^b^	1.67 ± 0.08 ^b^
Blue LED	24.89 ± 1.51 ^a^	2.07 ± 0.13 ^a^
Red LED	18.36 ± 0.15 ^c^	1.69 ± 0.05 ^b^

Means of ± standard errors followed by different letters within columns were significantly different according to Duncan’s multiple range test with one-way ANOVA; *n* = 6.

**Table 5:** Sucrose, starch, total phenolic content, and total flavonoid content of *H. corymbosa* (L.) Lam leaves after 4 weeks of being grown under different light sources at the same light intensity of 100 µmol·m^−2^·s^−1^.

Light Source	Content (mg/g _FW_)			
	Sucrose	Starch	Total Phenolic	Total Flavonoid
Fluorescent lamp	25.85 ± 3.64 ^a^	80.01 ± 5.45 ^b^	1.56 ± 0.07 ^c^	0.68 ± 0.06 ^b^
Blue LED	18.38 ± 1.08 ^b^	101.31 ± 6.56 ^a^	1.90 ± 0.08 ^b^	1.48 ± 0.30 ^a^
Red LED	17.69 ± 1.45 ^b^	78.04 ± 8.20 ^b^	3.50 ± 0.16 ^a^	0.58 ± 0.08 ^b^

Means of ± standard errors followed by different letters within columns were significantly different by Duncan’s multiple range test with one-way ANOVA; *n* = 6.

## Data Availability

The data presented in this study are available on request from the corresponding author. The data are not publicly available due to privacy.
